# Lenvatinib Plus Programmed Cell Death Protein-1 Inhibitor Beyond First-Line Systemic Therapy in Refractory Advanced Biliary Tract Cancer: A Real-World Retrospective Study in China

**DOI:** 10.3389/fimmu.2022.946861

**Published:** 2022-07-27

**Authors:** Changying Shi, Yulong Li, Cheng Yang, Liang Qiao, Liukang Tang, Yuting Zheng, Xue Chen, Youwen Qian, Jiamei Yang, Dong Wu, Feng Xie

**Affiliations:** ^1^ Department of Liver Surgery I, Eastern Hepatobiliary Surgery Hospital, Affiliated to Naval Medical University, Shanghai, China; ^2^ Department of Biliary Tract Surgery III, Eastern Hepatobiliary Surgery Hospital, Affiliated to Naval Medical University, Shanghai, China; ^3^ Department of Special Treatment, Eastern Hepatobiliary Surgery Hospital, Affiliated to Naval Medical University, Shanghai, China; ^4^ Department of Radiology, Eastern Hepatobiliary Surgery Hospital, Affiliated to Naval Medical University, Shanghai, China; ^5^ Department of Pathology, Eastern Hepatobiliary Surgery Hospital, Affiliated to Naval Medical University, Shanghai, China

**Keywords:** lenvatinib, PD-1 inhibitor, immunotherapy, target therapy, biliary tract cancer, second-line agents

## Abstract

**Background:**

Currently, no second-line systemic treatment regimen has been recommended in advanced biliary tract cancer (BTC). Cumulative clinical evidence showed that systemic treatment with tyrosine kinase inhibitors (TKIs) in combination with immunotherapy may shed light on the dim clinical outcome in advanced BTC.

**Objective:**

The aim of this study is to evaluate the anticancer efficacy of lenvatinib plus programmed cell death protein-1 (PD-1) antibody in patients with BTC who progressed after first-line cisplatin/gemcitabine (CisGem) chemotherapy.

**Methods:**

Patients with advanced BTCs who progressed after CisGem were recruited. A combination regimen of lenvatinib (8/12 mg daily) plus PD-1 antibody (200/240 mg injection every 3 weeks) was prescribed. Clinicopathological information and therapeutic outcome, including tumor subtypes, biomarkers, treatment duration, adverse events (AE), progression-free survival (PFS), and overall survival (OS), were recorded and estimated.

**Results:**

A total of 351 patients with BTCs were reviewed and 74 were recruited eventually: 35 had intrahepatic cholangiocarcinoma (47.3%), 4 had extrahepatic cholangiocarcinoma (5.4%), and 35 had gallbladder cancer (47.3%). The median administered cycles of PD-1 antibody were 6.43 (95% CI: 5.83–7.04) cycles, and the median duration of lenvatinib medication was 21.0 weeks (95% CI: 18.04–23.93). Twenty-eight patients (37.83%) experienced detectable objective response per RECIST1.1 within a median follow-up duration of 15.0 months. The objective response rate (ORR) was 20.27% (95% CI: 10.89%–29.65%), and the disease control rate (DCR) was 71.62% (95% CI: 61.11%–82.14%). The median PFS and OS were 4.0 months (95% CI: 3.5–5.0) and 9.50 months (95% CI: 9.0–11.0), respectively. Seventy-three patients (98.64%) reported AEs and 39 (52.70%) experienced ≥grade 3 AEs. In subgroup analyses, tumoral PD-L1 expression ≥50% and tumor mutation burden (TMB) ≥2.5 Muts/Mb were associated with prolonged PFS.

**Conclusion:**

Lenvatinib plus PD-1 antibody treatment shows an active trend towards improving survival in patients with advanced BTCs after failure with CisGem chemotherapy. The treatment-related AEs are worthy of attention and are manageable.

## Introduction

The incidence of biliary tract cancer (BTC), formerly considered rare, increased significantly in the last two decades globally (1). Although increasing types of biological agents and immune-oncology regimens emerged in hepatocellular carcinoma, there are limited therapies available in advanced BTC. Cisplatin/gemcitabine (CisGem)-based chemotherapy is currently recommended as the standard first-line therapy in advanced BTCs, although both its efficacy and tolerance are suboptimal (2). In the recent ABC-06 study, FOLFOX (folinic acid, fluoroutacil, and oxaliplatin) was evaluated as a second-line treatment after progression with CisGem (3). It demonstrated only a modest 1-month survival benefit against best supportive care. This frustrating result prompts novel effective therapeutic strategies to be tested so as to qualify as a second- or above-line therapy.

Although immunotherapy has revolutionized the treatment standard of several hematological and solid malignancies, its role in advanced BTC is still unclear. Monotherapy with immune checkpoint inhibitors (ICIs) in advanced BTC has presented conflicting results, suggesting further investigation in agent combination and deeper insight into subgroup selection.

Lenvatinib is an inhibitor of receptor tyrosine kinases, targeting vascular endothelial growth factor receptors (VEGFR1–3), fibroblast growth factor receptors (FGFR1–4), KIT, and RET (4). Owing to its capability of inhibiting multiple kinases in nanomole concentration, lenvatinib is now broadly used in the treatment of a variety of solid cancers, including differentiated thyroid cancer, hepatocellular carcinoma, and renal cell carcinoma, as a single agent or in combination with another drug (5). Several preliminary assessments of lenvatinib monotherapy or combination therapy with ICIs as first- or non-first-line therapy were reported, but the results were suboptimal and need further validation (6–9). Hereby, we reported a single arm of patients with refractory advanced BTCs, treated with lenvatinib plus programmed cell death protein-1 (PD-1) antibody as a second- or above-line systemic therapy.

## Materials and methods

### Study Design and Patients

This was a single-center retrospective study assessing the efficacy and safety of TKI lenvatinib associated with PD-1 antibody as a systemic therapy beyond 1st-line after the failure of CisGem chemotherapy at a hepatobiliary specific referral center (Eastern Hepatobiliary Surgery Hospital). BTC patients who received lenvatinib plus PD-1 antibody synchronously or successively as a second- or above-line systemic therapy from January 1, 2019 to March 31, 2021 were reviewed. This study was approved by the Institutional Ethics Committee of Shanghai Eastern Hepatobiliary Surgery Hospital. The study protocol conformed to the principles of the Declaration of Helsinki. The statistical analysis was conducted according to the intention-to-treat principle. All the data were updated and censored on February 28, 2022.

The patients with advanced BTCs who experienced progression after CisGem in first-line therapy were permitted to enroll. Advanced BTC was defined as initially diagnosed unresectable BTC (pathologically proved by biopsy or surgical specimen, multiple lesions, extrahepatic metastasis, and less future remnant liver) or relapses after surgery. Other eligibility criteria included good physical status with an Eastern Co-operative Oncology Group (ECOG) performance status score of 0–2, a Child–Pugh score of 5–6, and no severe comorbidities. The patients previously treated with other chemotherapy regimens or immunotherapies were excluded. Detailed information of the clinical protocol was explained to each patient, and the written informed consent forms were collected.

### Treatment Protocol

Patients were prescribed to orally take lenvatinib mesilate capsules (Patheon Inc.) 12 mg/day for body weight ≥ 60 kg or 8 mg/day for body weight < 60 kg as standard. To avoid acute intolerable side effects caused by lenvatinib from the start, a stepwise manner was undertaken. Patients were encouraged to take a reduced dose from 8 mg/day (≥60 kg) or 4 mg/day (<60 kg) for a week before reverting to the standard dose on day 8. Those who developed adverse events (AEs) related to lenvatinib had their dose reduced, or had their medication interrupted or discontinued depending on the severity. The PD-1 antibody was intravenously administered (200 mg of sintilimab or tislelizumab or 240 mg of nivolumab or toripalimab) in a 3-week cycle. The medication would not be halted unless disease progression (PD) or ≥grade 3 treatment-related adverse event (TRAE) occurred.

### Response Assessment

Clinical information and laboratory data prior to initial medication from eligible patients were collected. Tumor evaluation was conducted based on computed tomography (CT) or magnetic resonance imaging (MRI). Response evaluation criteria in solid tumor (RECIST1.1) and immunotherapy-related RECIST (irRECIST) were utilized to evaluate tumor response (10–12). The investigators and a panel of independent radiologists evaluated the images separately. Any discrepancy, mainly regarding lymph node enlargement-triggered PD and irRECIST-related partial response (PR)/stable disease (SD), was discussed and combined. The objective response rate (ORR) was defined as the proportion of patients with complete response (CR) or PR of total evaluated. The disease control rate (DCR) was defined as the proportion of patients with CR, PR, and SD. Overall survival was calculated from the date of medication initiation until the date of death. Progression-free survival (PFS) was measured from the date of medication initiation until the date of disease progression or death.

### Safety Evaluation and Quality of Life

Safety was continuously evaluated every 4 weeks by manifestation and laboratory tests, including hemogram, liver function, thyroid function, and myocardial enzyme. TRAEs were recorded according to National Cancer Institute Common Terminology Criteria for Adverse Events version 5.0 (CTCAE 5.0) (13). The quality of life (QOL) was assessed with ECOG score (14). A rising score from baseline to 3 or higher was regarded as a significant disturbance to QOL.

### Histological Biomarker Assessment

PD-L1 expression and tumor mutation burden (TMB) were investigated as potential biomarkers in this study. Immunohistochemistry was performed to determine the expression of PD-L1 using E1L3N (PD-L1 XP Rabbit mAb, Cell Signaling Technology, Danvers, USA) on tumor biopsy samples. Samples with 50% or more tumor cells for PD-L1 exhibiting linear cell membranous staining were considered positive (15, 16). The TMB was determined using next-generation sequencing (NGS, Illumina nova seq) (17). Genomic alterations including base substitutions, insertions, deletions, gene rearrangement, and fusions were analyzed to form mutation load according to the megabase (Mb) (Integrated DNA Technologies, USA).

### Statistical Analysis

The continuous and categorical variables were calculated with the appropriate method including the Student’s *t*-test, the Mann–Whitney *U*-test, the Chi-square test, or Fisher’s exact test. The Kaplan–Meier method was employed to estimate the PFS and OS, and to accomplish the survival comparison in subgroups. The statistical analyses were performed with SPSS 21.0 for Windows (SPSS, Chicago, IL, USA) and R software 4.0.2 (R Foundation for Statistical Computing, Vienna, Austria). A *p*-value < 0.05 was considered statistically significant.

## Results

### Patients’ Baseline Characteristics

**Figure 1:**
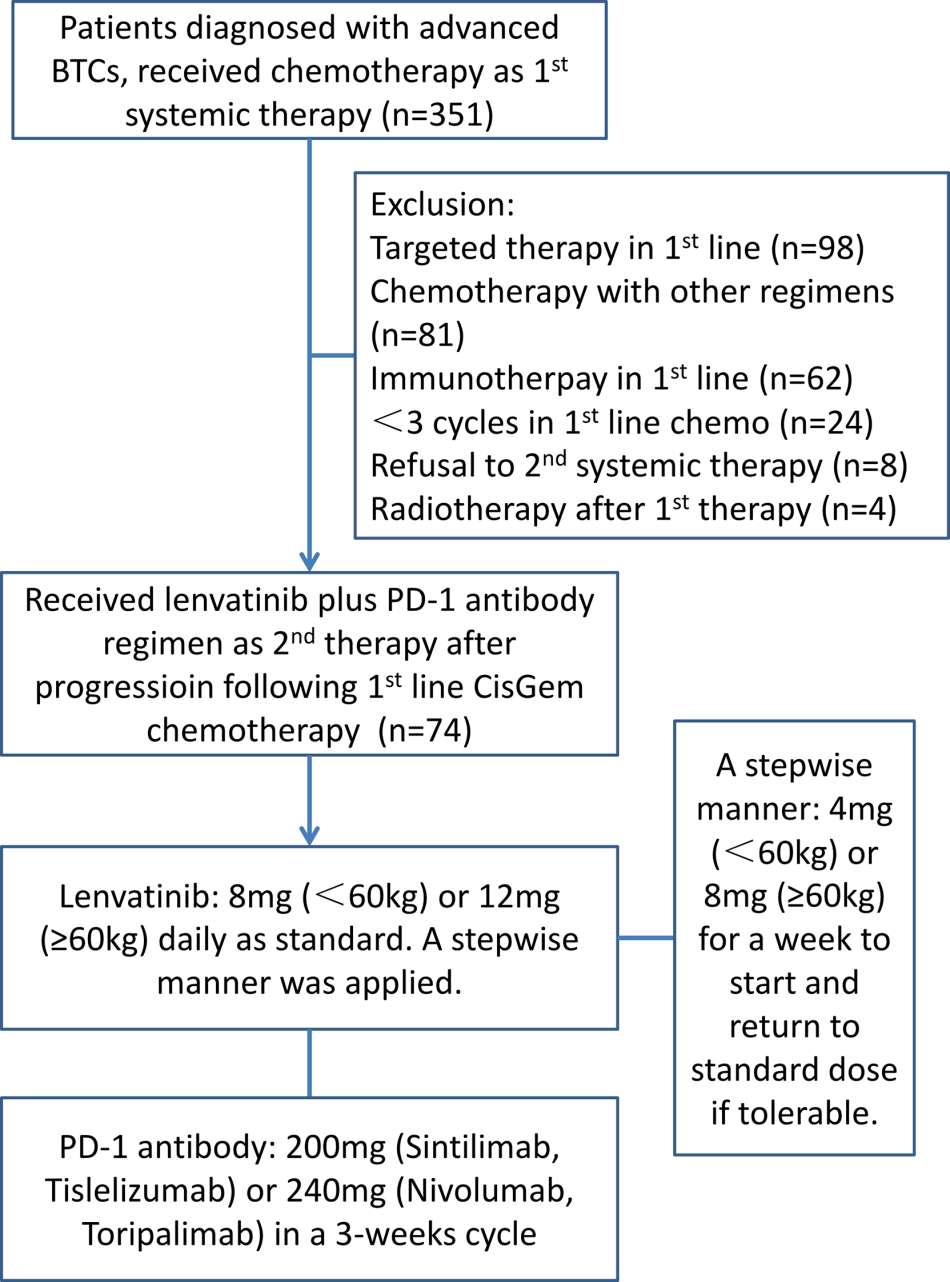
The flowchart of the study illustrates the enrollment procedure and the treatment protocol.

A total of 351 patients diagnosed with BTC were reviewed and 74 patients were recruited. All the patients were willing to attend the systemic therapy. The flowchart of the study and the treatment protocol is shown in **Figure 1**. The cohort included 35 (47.3%) with intrahepatic cholangiocarcinoma (iCCA), 4 (5.4%) with extrahepatic cholangiocarcinoma (eCCA), and 35 (47.3%) with gallbladder cancer (GBC), with a male/female ratio of 1.55 (45/29). The median age was 62.5 years (range: 43–78). In etiology surveillance, 24 patients (32.43%) had background liver diseases, which included 23 (31.08%) hepatitis B virus infections. Most patients had good physical performance except for two patients who got an ECOG PS score of 2. Twenty-five patients had received local regional therapy previously, including surgery (11, 14.86%), radiotherapy (11, 14.86%), and TACE (3, 4.05%). Forty-four patients (59.46%) had extrahepatic metastases. The baseline patient demographics and clinical characteristics are summarized in **Table 1**.

**Table 1:** Baseline demographics.

Parameters	Subjects (N=74)
Gender, *n* (%)	
Male	45 (60.8%)
Female	29 (39.2%)
Age years, (median, range)	62.5 (43–78)
Age group	
<60 years	26 (35.1%)
≥60	48 (46.9%)
ECOG performance, *n* (%)	
0	33 (44.6%)
1	39 (52.7%)
2	2 (2.7%)
Tumor subtype, *n* (%)	
Intrahepatic cholangiocarcinoma	35 (47.3%)
Extrahepatic cholangiocarcinoma	4 (5.4%)
Gallbladder cancer	35 (47.3%)
Background liver diseases, *n* (%)	24 (32.4%)
Hepatitis B virus infection, *n* (%)	23 (31.1%)
Previous local regional therapy, *n* (%)	
Surgery	11 (14.9%)
Radiation	11 (14.9%)
Transarterial chemo-emblization	3 (4.1%)
Child–Pugh score, *n* (%)	
5	62 (83.8%)
6	20 (13.5%)
7	2 (2.7%)
CA19-9, *n* (%)	
<500 μg/L	27 (36.5%)
≥500 μg/L	47 (63.5%)
Extrahepatic metastasis, *n* (%)	
Yes	30 (40.5%)
Measurable lesions burden, *n* (%)	
<3	54 (73.0%)
≥3	20 (27.0%)
TNM stage	
T_3_N_0_M_0_	22 (29.7%)
T_1-3_N_1_M_0_	27 (36.4%)
T_4_N_0-1_M_0_	6 (8.1%)
T_any_N_2 or any_M_0 or 1_	19 (25.6%)
White cell counts, (median×10^9^/L, Range)	7.10 (4.09–9.93)
Platelet counts, (median×10^9^/L, Range)	174.5 (57–299)
Prior chemotherapy cycles, *n* (%)	
<6 cycles	43 (58.1%)
≥6 cycles	31 (41.9%)
Line	
2nd line, *n* (%)	54 (73.0%)
3rd line, *n* (%)	17 (23.0%)
4th line, *n* (%)	3 (4.1%)

Data were presented as n (%) or median with range as appropriate.

### Treatment

The lenvatinib+PD-1 antibody regimen was administered as the 2nd-line systemic therapy in 54 patients (73.0%), the 3rd-line therapy in 17 (23.0%), and the 4th-line therapy in 3 (4.1%). The usage of PD-1 antibody injection included nivolumab (6.8%), sintilimab (51.4%), toripalimab (24.3%), and tislelizumab (17.6%). The median administered cycles of PD-1 antibody were 6.43 cycles (95% CI: 5.83–7.04), and all patients received at least 2 shots. The median duration of lenvatinib intake was 21.0 weeks (95% CI: 18.04–23.93), and all patients took lenvatinib for at least 4 weeks. Twenty-two patients were given reduced lenvatinib dosage in case of intolerable AEs. Sixty-three patients discontinued treatment owing to tumor progression, 5 discontinued due to intolerable AEs, and 6 patients remained under medication before the cutoff date.

### Efficacy

**Figure 2:**
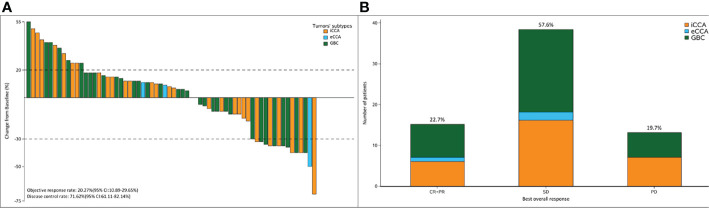
**(A)** The maximum of change of the sum of the target lesions; **(B)** best overall response per RECIST1.1 according to the tumor subtypes. The ORR and DCR were 22.7% and 71.62%, respectively.

Within a median follow-up duration of 15.0 months (95% CI: 12.874–17.126), 66 (89.19%) patients were available for efficacy assessment. Twenty-eight patients (37.83%) experienced detectable objective response per irRECIST, while 38 patients showed augmentation of measurable tumors. **Figure 2** shows the maximum change of the sum of measurable lesions and the best overall response.

**Figure 3:**
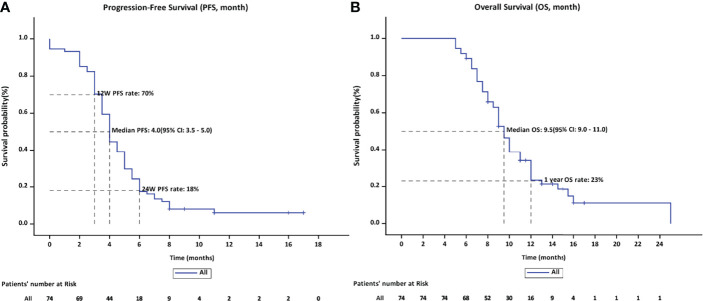
**(A)** The PFS rate on 12 weeks and 24 weeks were 70% and 18%, respectively, with a median PFS of 4.0 months. **(B)** The median OS was 9.5 months (95% CI: 9.0–11.0) and the OS rate of 1 year was 23%.

**Figure 4:**
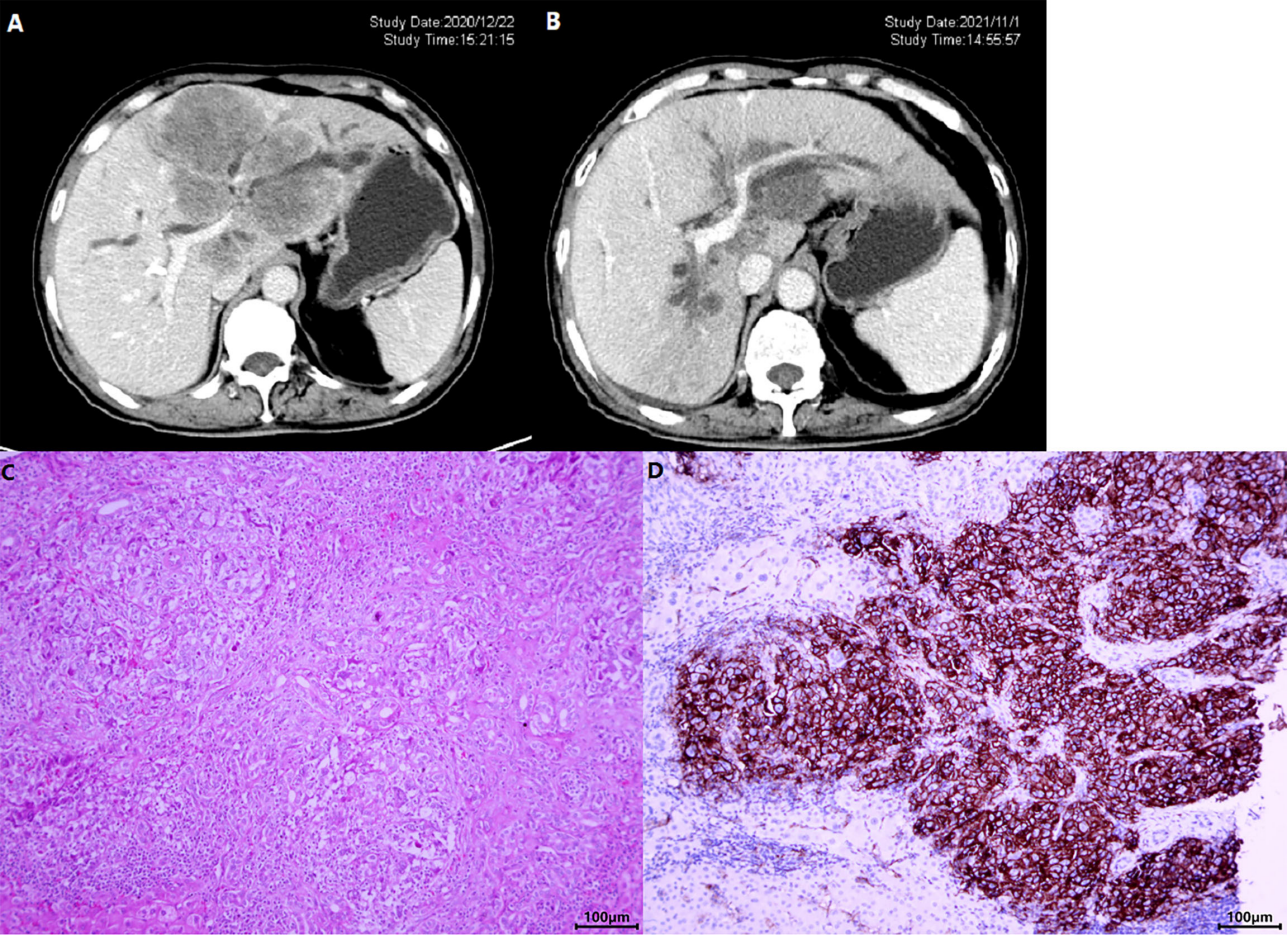
A patient with iCCA received lenvatinib+sintilimab as 2nd-line systemic therapy. The CT images before **(A)** and after **(B)** nearly 1-year therapy showed a significant shrinkage of the target lesion, which was mainly located in the left hemi-liver and invaded the left branch of portal vein. **(C)** Shows the HE staining of the tumor and **(D)** displays the photomicrographs of immunohistochemistry staining of PD-L1 expression.

The ORR following lenvatinib+PD-1 antibody treatment as the 2nd- and above-line systemic therapy in advanced BTCs was 20.27% (95% CI: 10.89%–29.65%), with 0 CR (0%) and 15 (20.27%) PRs. Thirty-eight patients achieved stable disease, and DCR was 71.62% (95% CI: 61.11%–82.14%) (**Table 2**). The median PFS was 4.0 months (95% CI: 3.5–5.0), and the PFS rate at 12 weeks was 70.0% (**Figure 3A**). The median OS was 9.50 months (95% CI: 9.0–11.0) and 1-year OS rate was 23% (**Figure 3B**). **Table 2** displays the detailed information of the therapeutic responses. Five patients experienced a deep regression in tumor size and did not progress until the censored follow-up date (**Figures 4A, B**). The ORR in iCCA, eCCA, and GBC was 20.69% (95% CI: 5.01%–36.4%), 33.33% (95% CI: −110%–177%), and 23.53% (95% CI: 8.51%–38.6%), respectively. The DCR in iCCA, eCCA, and GBC was 75.86% (95% CI: 59.3%–92.4%), 100%, and 82.35% (95% CI: 68.9%–95.9%), respectively (**Table 3**).

**Table 2:** Treatment summary and therapeutic responds.

Category	Outcome
Lenvatinib regimen duration (weeks, median, range)	18.5 (6–69)
PD-1 cycles (*n*, median, range)	6 (2–14)
Complete response (CR, *n*, %)	0 (0%)
Partial response (PR, *n*, %)	15 (20.3%)
Stable disease (SD, *n*, %)	38 (51.4%)
Progression disease (PD, *n*, %)	13 (17.6%)
ORR	20.27% (95% CI: 10.89%–29.65%)
DCR	71.62% (95% CI: 61.11%–82.14%)

PD-1, programmed cell death protein-1; ORR, objective response rate; DCR, disease control rate.

**Table 3:** Objective response rate/Disease control rate.

Tumor subtype	ORR	DCR
**iCCA**	20.69% (95% CI: 5.01%–36.4%)	75.86% (95% CI: 59.3%–92.4%)
**eCCA**	33.33% (95% CI: −110%–177%)	100%
**GBC**	23.53% (95% CI: 8.51%–38.6%)	82.35% (95% CI: 68.9%–95.9%)

ORR, objective response rate; DCR, disease control rate; iCCA, intrahepatic cholangiocarcinoma; eCCA, extrahepatic cholangiocarcinoma; GBC, gallbladder cancer.

### Tolerability and Safety

**Figure 5:**
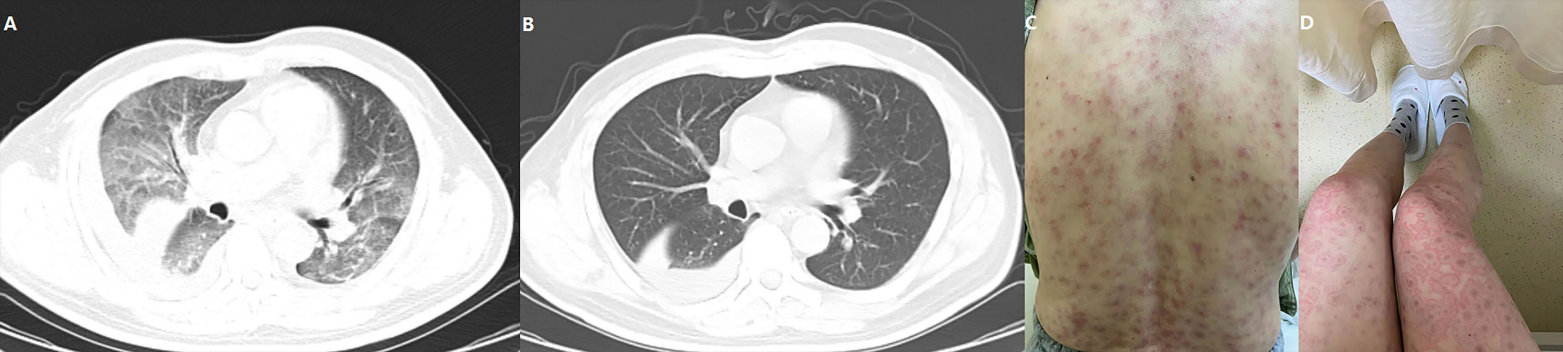
One patient experienced a grade 4 immune-related pneumonitis **(A)** and recovered following corticosteroid injection **(B)**. Another patient developed severe erythema that affected more than 80% of the skin area (**C**: back; **D**: thigh and legs), but the inner environment was not bothered.

In total, 73 patients (98.64%) reported AEs and 39 (52.70%) experienced ≥grade 3 AEs. The most common AEs were decreased appetite (81.08%), fatigue (31.08%), elevated aspartate aminotransferase (27.03%), hypertension (21.62%), and diarrhea (20.27%). Detailed information of AEs is shown in **Table 4**. Most patients were advised to continue taking the medication through reduced dosage or to have medical support. Five patients (6.76%) withdrew from treatment due to intolerable AEs, which included 1 grade 3 diarrhea, 1 grade 3 increased aspartate aminotransferase, 1 grade 4 immune-associated pneumonitis (**Figures 5A, B**), 1 grade 3 immune-related erythema (**Figures 5C, D**) and 1 grade 2 immune-associated myocarditis. Patients with immune related AEs (irAE) were treated with low-dose corticosteroids and recovered. The ECOG score increased from 0/1 to 2 in 42 (56.75%) patients after at least 4 weeks of treatment and caused a disturbance to QOL.

**Table 4:** Adverse events ranking.

Events	Any AE (*n*, %)	Grade 1–2 AEs (*n*, %)	≥Grade 3 AEs (*n*, %)
Total	73 (98.64%)	60 (81.08%)	39 (52.70%)
Decreased appetite	60 (81.08%)	56 (75.68%)	13 (17.57%)
Fatigue	23 (31.08%)	23 (31.08%)	7 (9.46%)
Elevated aspartate aminotransferase	20 (27.03%)	16 (21.62%)	4 (5.41%)
Hypertension	16 (21.62%)	14 (18.92%)	8 (10.81%)
Diarrhea	15 (20.27%)	10 (13.51%)	5 (6.76%)
Abdominal pain	11 (14.86%)	10 (13.51%)	1 (1.35%)
Nausea	10 (13.51%)	10 (13.51%)	-
Palmar plantar erythrodysesthesia syndrome	9 (12.16%)	9 (12.16%)	-
Thrombocytopenia	9 (12.16%)	9 (12.16%)	-
Anemia	9 (12.16%)	8 (10.81%)	1 (1.35%)
Headache	8 (10.81%)	8 (10.81%)	-
Erythema	7 (9.46%)	7 (9.46%)	2 (2.70%)
Proteinuria	6 (8.11%)	6 (8.11%)	-
Hypothyroidism	5 (6.76%)	5 (6.76%)	-
Myalgia	4 (5.41%)	4 (5.41%)	-
Alopecia	3 (4.05%)	3 (4.05%)	-
Immune-associated pneumonitis	2 (2.70%)	1 (1.35%)	1 (1.35%)
Immune-associated myocarditis	1 (1.35%)	1 (1.35%)	-

AE, adverse event.

### Biomarkers

The spider diagram illustrated the serum CA19-9 concentration change in treatment duration (**Supplementary Figure 1A**). The change flow was well correlated to the tumor regression and progression accordingly with an area under the curve of 0.554 (**Supplementary Figure 1B**).

**Figure 6:**
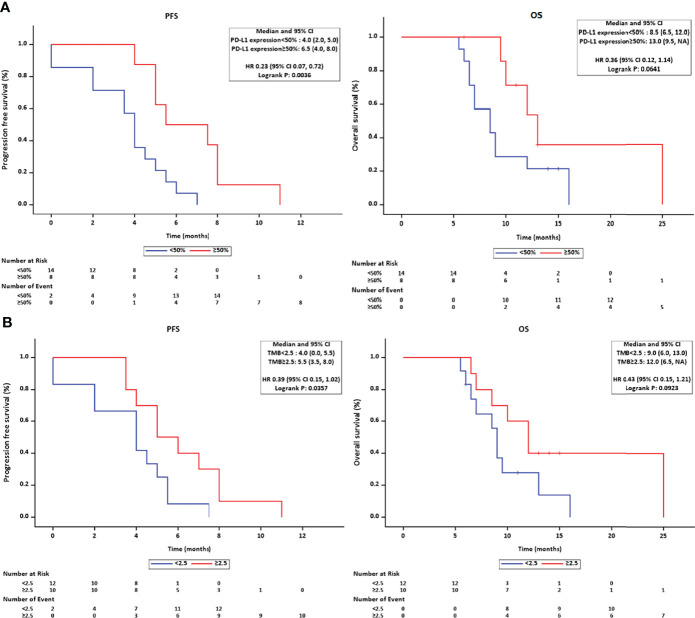
**(A)** The Kaplan–Meier method estimated the survival length in PD-L1-positive/-negative subgroups and found a marginal superiority for RFS in the PD-L1 positive group. **(B)** A significant longer PFS was observed in the higher TMB subgroup.

The genomic profile of PD-L1 expression and TMB was available in 22 patients. Tumoral PD-L1 staining (**Figures 4C, D**) was positive (≥50% cells as cutoff) in 8 patients (36.36%), 6 of whom (75.0%) achieved objective response and all patients had their disease controlled. Compared with negative expression, patients with tumoral PD-L1 expression presented prolonged PFS (5.50 months, 95% CI: 2.035–8.965 vs. 4.00 months, 95% CI: 3.414–4.586; *p =* 0.004), but not OS (13.00 months, 95% CI: 9.741–16.259 vs. 8.50 months, 95% CI: 5.778–11.222; *p =* 0.064) (**Figure 6A**).

The cutoff value of TMB was set at 2.5 Muts/Mb according to the average value released on The Cancer Genome Atlas (TCGA) website. Ten patients had higher TMB (≥2.5 Muts/Mb), and subgroup survival analysis revealed a prolonged PFS (5.00 months, 95% CI: 2.934–7.066, *p =* 0.036) in the higher TMB group, but not OS (12.0 months, 95% CI: 8.964–15.036, *p =* 0.092) (**Figure 6B**).

## Discussion

This study evaluated the efficacy and safety of the two-drug regimen, lenvatinib plus PD-1 inhibitor, as a second- or above-line systemic therapy in refractory advanced BTCs. The results showed an ORR of 20.27% (95% CI: 10.89%–29.65%) and a DCR of 71.62% (95% CI: 61.11%–82.14%), with a median OS of 9.5 months. A total of 98.64% patients developed any-grade AEs and 52.70% developed grade 3/4 AEs. The results were similar to those of recent studies involving a single agent and combined regimens treating refractory advanced BTCs (7, 15, 18–21). This combination regimen prolonged the survival duration in both second- and above-line systemic therapies, without significant difference (*p =* 0.809). Additionally, this study found that 56.75% of the population got a worse ECOG physical score, which partly played a negative role in ensuring patients’ compliance.

The long-term survival of patients with BTCs was dismal, with 5-year survival rates of 10%–50% (1, 21). ABC-01 and ABC-02 clinical trials established the standard of CisGem as a first-line systemic therapy in local advanced and metastatic BTCs (2, 22). The ABC-06 trial explored the effectiveness of the FOLFOX regimen as a second-line chemotherapy after progression (3). This study reported a median OS, 6-month survival rate, and 12-month survival of 6.2 months, 50.6%, and 25.9%, respectively, in the FOLFOX plus active symptom control population, which showed a significant improvement in survival compared with the active symptom control group. However, it also reported relatively high AE rates, with a 52% incidence rate of grade 3–5 AEs. Three patients died due to chemotherapy-related adverse effects. Considering its limited survival benefit and high adverse effects, the FOLFOX regimen might not be an ideal second-line therapy in refractory advanced BTCs.

Efforts were made to find potential targeted strategies in treating BTCs, including multiple pathways like angiogenesis, human epidermal growth factor receptor family, and other actionable targets (23, 24). Several existing and emerging molecular targeted agents were tested as second-line therapy. In the REACHIN study, 66 patients diagnosed with BTC who had already progressed to CisGem chemotherapy were randomized in a phase II study to receive regorafenib or placebo (25). Although the regorafenib group showed an improved PFS versus the placebo group (3.0 months, 95% CI: 2.3–4.9 vs. 1.5 months, 95% CI: 1.2–2.0, *p =* 0.004), no patients reached objective response and no survival benefit was found regarding OS. The result showed that the addition of sorafenib to gemcitabine did not demonstrate improved efficacy in advanced BTC patients. Recently, the efficacy and safety of lenvatinib in treating advanced BTC were evaluated in a single-arm study (18). Forty-one patients with histologically confirmed BTCs received 8 mg (weight < 60 kg) or 12 mg (weight ≥ 60 kg) of lenvatinib orally per day. The ORR was 12%, with a median PFS of 3.8 months and an OS of 11.4 months. Up to 95.1% patients in total experienced TRAEs. In a phase II study of lenvatinib monotherapy as a 2nd-line treatment, the ORR was 11.5% and the ≥grade 3 AEs occurred in 80.8% of total patients (20). The median PFS and median OS were 3.19 months per investigator assessment and 7.35 months, respectively. In another study in China, with pembrolizumab combined with lenvatinib as a non-first-line therapy, the ORR and the DCR were 25% and 78.1%, respectively. The median PFS and median OS were 4.9 months and 11.0 months, respectively (7). Our results on lenvatinib plus PD-1 antibody showed a close survival benefit with an mPFS of 4.0 months and an mOS of 9.5 months. This indicated that accumulated clinical practices would probably pave the way to expand the usage of targeted agents combined with ICIs in advanced BTCs. There are several ongoing clinical trials regarding the lenvatinib plus ICIs combination regimen. **Supplementary Table 1** summarizes the ongoing trials registered on clinicaltrials.gov.

Among patients who progressed from the first-line CisGem regimen, their AE experiences in chemotherapy may probably be an obstacle to achieve good compliance in subsequent treatments, especially in a TKI/PD-1 antibody combination regimen. The two-drug pembrolizumab plus lenvatinib regimen reportedly obtained a 100% and 59.3% rate of any-grade AEs and ≥grade 3 AEs, respectively (7). In a systematic review evaluating the safety and efficacy of pembrolizumab plus lenvatinib in cancers, ≥grade 3 AEs occurred in 68.0% of all patients (26). Our result showed a 98.64% occurrence rate of any-grade AEs and a 48.65% occurrence rate of ≥grade 3 AEs, even under a stepwise manner. The treatment was called off in five patients due to intolerable AEs. More than half of the patients (56.75%) reported a decline in QOL related to the treatment, which should not be neglected.

The prognostic value of the tumoral expression of PD-L1 and a higher TMB in molecular targeted therapy and immunotherapy were not validated. Korean researchers reported a 71% positive (defined as ≥1% cells stained) rate of PD-L1 in BTCs, and the ORR with pembrolizumab treatment was improved in the PD-L1 expression ≥ 50% subgroup (16). In another study, KRAS alteration and chromosomal instability tumors were associated with resistance to immunotherapy, and the majority of patients (95.0%) with these resistance factors showed no clinical benefit to PD-1/PD-L1 blockade and harbored low TMB (27). Germline or somatic mutations in DNA damage repair (DDR) genes were found in 63.5% of patients with BTC and were significantly associated with longer survival while receiving first-line platinum-containing chemotherapies (28). Our study, with a limited sample size, showed a probability of survival benefit regarding PFS in tumoral PD-L1 expression ≥50% or in TMB ≥2.5 Muts/Mb. Cumulative research indicated that BTCs held scores of mutational varieties in genomic profiling. It is still a long way to go in finding genomic prognostic biomarkers in BTC.

This study has its share of limitations. Firstly, this study was retrospectively designed with a relatively small sample size, which might contribute to the sample bias. Secondly, the contribution of the three different anatomical locations derived from BTCs was not balanced, and a subtype analysis was not available due to the limited sample size. A relatively small proportion of patients with advanced eCCAs received systemic therapy owing to a constantly uncompensated liver function caused by biliary tract obstruction. This might be the reason why only three patients with eCCA were enrolled in this study. Lastly, a marginal survival benefit was detected in the PD-L1-positive or high TMB profile subgroup. Further investigation is necessary due to the limited sample size.

## Conclusion

Lenvatinib plus PD-1 blockade played an active role in the treatment of patients with advanced refractory BTCs who progressed following CisGem chemotherapy. A moderate proportion of treatment-related AEs could not be neglected in practice, though they could be treated with further observation and care.

## Data Availability Statement

The data presented in the study are deposited in the Sequence Read Archive (SRA), https://www.ncbi.nlm.nih.gov/sra. The accession number is PRJNA857805.

## Ethics Statement

The studies involving human participants were reviewed and approved by the Institutional Ethics Committee of Shanghai Eastern Hepatobiliary Surgery Hospital. The patients/participants provided their written informed consent to participate in this study.

## Author Contributions

SC, XF, WD and YJ designed this study. LY, YC, QL, TL,XC, YQ and ZY collected the data and perform the follow-up. SC, LY, and YC performed or supervised analyses. SC, WD, and XF interpreted the results. SC, LY and YC wrote the original manuscript. WD and XF revised the manuscript. All authors reviewed the manuscript and approved the final version submitted.

## Funding

This research has received funding from the Shanghai Municipal Health Commission Program (202140362), the Natural Science Foundation of Shanghai (Grant No. 16ZR1449200), and the Shanghai Shen Kang Hospital Development Center Planning (Grant No. SHDC12017X14).

## Conflict of Interest

The authors declare that the research was conducted in the absence of any commercial or financial relationships that could be construed as a potential conflict of interest.

## Publisher’s Note

All claims expressed in this article are solely those of the authors and do not necessarily represent those of their affiliated organizations, or those of the publisher, the editors and the reviewers. Any product that may be evaluated in this article, or claim that may be made by its manufacturer, is not guaranteed or endorsed by the publisher.
