# Supplementary material for: Lenvatinib Plus Programmed Cell Death Protein-1 Inhibitor Beyond First-Line Systemic Therapy in Refractory Advanced Biliary Tract Cancer: A Real-World Retrospective Study in China
Source: Front Immunol. 2022 Jul 27;13:946861. doi: 10.3389/fimmu.2022.946861 (PMC9364266; doi:10.3389/fimmu.2022.946861)

Supplemental table 1. Undergoing clinical trials investigating Lenvatinib+ICIs in previous treated advanced BTCs

|  | Title | Status | Study Results | Conditions | Interventions | Location |
| --- | --- | --- | --- | --- | --- | --- |
| 1 | Toripalimab Plus Lenvatinib as Second-line Treatment in Advanced Biliary Tract Cancers | Recruiting | No Results Available | Advanced Biliary Tract Cancer | Drug: Toripalimab plus Lenvatinib | Chinese Academy of Medical Sciences & Peking Union Medical College Hospital, Beijing, Beijing, China |
| 2 | Lenvatinib Plus Paclitaxel for Patients With Advanced Biliary  Tract Cancer Who Failed to Gemcitabine-based Treatment | Not yet recruiting | No Results Available | Advanced Biliary Tract Cancer | Drug: Lenvatinib Pill,Paclitaxel | Taiwan Cooperative Oncology Group, National Health Research  Institutes, Taipei, Taiwan |
| 3 | Study of Lenvatinib (E7080) in Unresectable Biliary Tract  Cancer (BTC) Who Failed Gemcitabine-based Combination  Chemotherapy | Completed | Has Results | Biliary Tract Cancer | Drug: Lenvatinib | Nagoya, Aichi, Japan; Kashiwa, Chiba, Japan; Yokohama, Kanagawa, Japan; Ina-machi, Saitama, Japan; Chuo-ku, Tokyo, Japan; Koto-ku, Tokyo, Japan; Mitaka, Tokyo, Japan |
| 4 | Lenvatinib in Patients With Previously Treated Advanced  Biliary Tract Cancer | Completed | No Results Available | Cholangiocarcinoma  Biliary Tract Cancer  Targeted Therapy | Drug: Lenvatinib | Chinese Academy of Medical Sciences & Peking Union Medical  College Hospital, Beijing, Beijing, China |
| 5 | Capecitabine Combined With Lenvatinib and Tislelizumab as  Adjuvant Treatment After Resection in Patients With BTC | Recruiting | No Results Available | Biliary Tract Cancer | Drug: Capecitabine combined with  Lenvatinib and tislelizumab | Fudan University Shanghai Cancer Center, Shanghai, China |
| 6 | Tislelizumab(Anti PD-1), Lenvatinib and GEMOX  Transformation in the Treatment of Potentially Resectable,  Locally Advanced Biliary Tract Cancer | Recruiting | No Results Available | Biliary Tract Cancer  PD-1 Antibody  Gemox  Lenvatinib | Drug: PD-1+Lenvatinib+GEMOX | Zhongshan hospital, Shanghai, China |
| 7 | Tislelizumab+Lenvatinib+Gemox Regiment for Potentially  Resectable Locally Advanced Malignant Tumors of Biliary  System | Not yet recruiting | No Results Available | Potentially Resectable Locally Advanced  Malignant Tumors of Biliary System | Malignant Tumors of Biliary System | Drug: Tislelizumab |
| 8 | Tisleizumab Combined With Lenvatinib and XELOX Regimen  (Oxaliplatin Combined With Capecitabine) in the First-line  Treatment of Advanced and Unresectable Biliary Tract  Tumors | Recruiting | No Results Available | Biliary Tract Tumor | Drug: Tislelizumab  Drug: Lenvatinib  Drug: Oxaliplatin  Drug: Capecitabine | Jiangsu Province Hospital, Nanjing, Jiangsu, China |

Supplemental figure 1. (A) The spider diagram recorded most patients’ serum CA19-9 change in the treatment duration. (B) The area under curve (AUC) for predicting OS by baseline CA19-9 level was 0.554, the optimal cut-off value was 340 (ug/L), and the sensitivity and specificity were 82.1% and 44.4%, respectively.


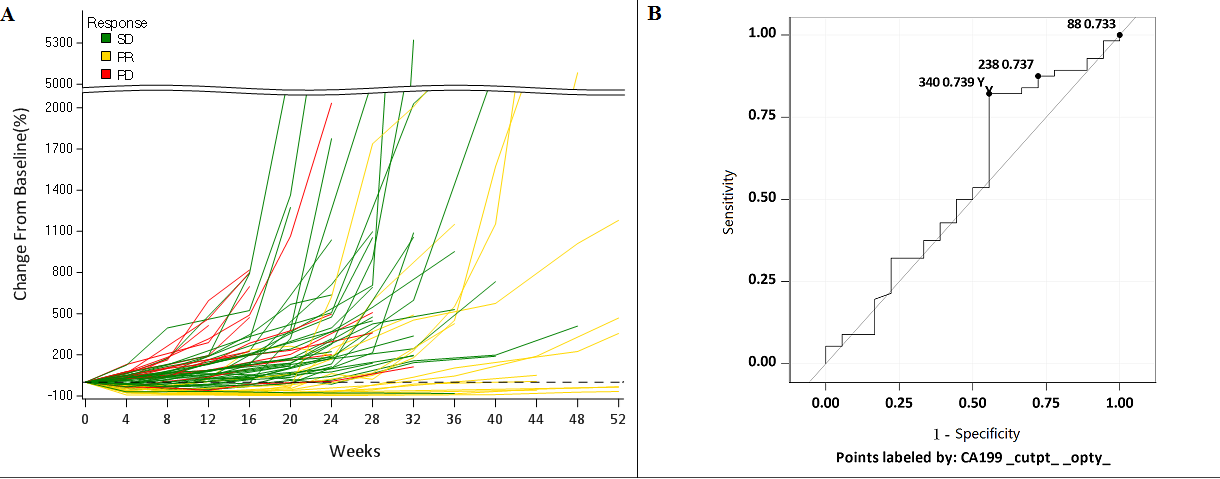

Supplement: Supplementary file 1 [file DataSheet_1.docx]
